# Dynamic Thromboembolic Risk Modelling to Target Appropriate Preventative Strategies for Patients with Non-Small Cell Lung Cancer

**DOI:** 10.3390/cancers11010050

**Published:** 2019-01-08

**Authors:** Marliese Alexander, David Ball, Benjamin Solomon, Michael MacManus, Renee Manser, Bernhard Riedel, David Westerman, Sue M. Evans, Rory Wolfe, Kate Burbury

**Affiliations:** 1 Department of Epidemiology and Preventive Medicine Monash University, 99 Commercial Road, Melbourne, VIC 3004, Australia; sue.evans@monash.edu (S.M.E.); rory.wolfe@monash.edu (R.W.); 2 Pharmacy Department, Peter MacCallum Cancer Centre, 305 Grattan St, Melbourne, VIC 3000, Australia; 3 Sir Peter MacCallum Department of Oncology, The University of Melbourne, Parkville, VIC 3010, Australia; david.ball@petermac.org (D.B.); ben.solomon@petermac.org (B.S.); michael.macmanus@petermac.org (M.M.); bernhard.riedel@petermac.org (B.R.); david.westerman@petermac.org (D.W.); 4 Department of Radiation Oncology Peter MacCallum Cancer Centre, 305 Grattan St, Melbourne, VIC 3000, Australia; 5 Department of Medical Oncology, Peter MacCallum Cancer Centre, 305 Grattan St, Melbourne, VIC 3000, Australia; 6 Department of Respiratory and Sleep Disorders Medicine, Royal Melbourne Hospital, Grattan Street, Parkville, VIC 3050, Australia; renee.manser@petermac.org; 7 Department of Anaesthesia, Perioperative and Pain Medicine, Peter MacCallum Cancer Centre, 305 Grattan St, Melbourne, VIC 3000, Australia; 8 Department of Pathology, Peter MacCallum Cancer Centre, 305 Grattan St, Melbourne, VIC 3000, Australia; 9 Department of Haematology, Peter MacCallum Cancer Centre, 305 Grattan St, Melbourne, VIC 3000, Australia

**Keywords:** thromboembolism, non-small cell lung cancer, risk prediction, pulmonary embolism, deep vein thrombosis

## Abstract

Prevention of cancer-associated thromboembolism (TE) remains a significant clinical challenge and priority world-wide safety initiative. In this prospective non-small cell lung cancer (NSCLC) cohort, longitudinal TE risk profiling (clinical and biomarker) was undertaken to develop risk stratification models for targeted TE prevention. These were compared with published models from Khorana, CATS, PROTECHT, CONKO, and CATS/MICA. The NSCLC cohort of 129 patients, median follow-up 22.0 months (range 5.6—31.3), demonstrated a hypercoagulable profile in >75% patients and TE incidence of 19%. High TE risk patients were those receiving chemotherapy with baseline fibrinogen ≥ 4 g/L and d-dimer ≥ 0.5 mg/L; or baseline d-dimer ≥ 1.5 mg/L; or month 1 d-dimer ≥ 1.5 mg/L. The model predicted TE with 100% sensitivity and 34% specificity (c-index 0.67), with TE incidence 27% vs. 0% for high vs. low-risk. A comparison using the Khorana, PROTECHT, and CONKO methods were not discriminatory; TE incidence 17–25% vs. 14–19% for high vs. low-risk (c-index 0.51–0.59). Continuous d-dimer (CATS/MICA model) was also not predictive of TE. Independent of tumour stage, high TE risk was associated with cancer progression (HR 1.9, *p* = 0.01) and mortality (HR 2.2, *p* = 0.02). The model was tested for scalability in a prospective gastrointestinal cancer cohort with equipotency demonstrated; 80% sensitivity and 39% specificity. This proposed TE risk prediction model is simple, practical, potent and can be used in the clinic for real-time, decision-making for targeted thromboprophylaxis. Validation in a multicentre randomised interventional study is underway (ACTRN12618000811202).

## 1. Introduction

Despite extensive clinical experience with safe, efficacious and cost-effective antithrombotic agents, thromboembolism (TE) remains a frequent and preventable complication among patients with cancer, with substantial adverse health and economic consequences [1,2,3,4].

Lung cancer remains the world’s most common and deadliest cancer [4]. Published data continues to demonstrate high TE rates (up to 34% in some cohorts) with a significant contribution to lung-cancer associated mortality and morbidity [5,6,7,8,9,10,11,12]. Furthermore, in terms of clinical impact, this burden is underestimated with the focus on clinically apparent macrovascular events—overlooking the haemostatic and endothelial dysfunction at a microvascular and tumour biology level, which contributes to morbidity, disease progression, and mortality. This interplay between haemostatic dysfunction and tumour cell survival, proliferation and metastatic spread [13], as well as the antiangiogenic and anti-tumour properties of anticoagulants [14,15], highlights additional opportunity for therapeutic impact. The key is identifying the appropriate patients at the appropriate time for greatest beneficial impact.

Pharmacologic thromboprophylaxis (P-TP) is a proven preventative and safe strategy, with up to 80% reduction in TE in cancer cohorts [16,17]. However, heterogeneity in TE risk and concerns regarding cumulative bleeding risk, has restricted “routine” P-TP utilisation within ambulatory patients [18,19,20]—where the majority of patients are managed and approximately 80% of TE events occur [5,21]. Clinicians are seeking simple but targeted guidance, for the appropriate application in high TE risk patients and time periods—and avoiding unnecessary intervention when TE risk is low. This requires understanding of both the heterogeneous and dynamic nature of the TE risk, across different cancer subtypes and anticancer therapies.

Published risk models for cancer-associated TE *(Khorana*, *CATS*, *PROTECHT*, *CONKO*, *and CATS/MICA)* [22,23,24,25,26] are limited by their design, potency, as well as complexity for real-time use in the clinic [27,28]. The Khorana Score (KS) [22] represents a weighted scoring system where high risk patients are defined by score ≥ 3 from baseline parameters: cancer type (2 points for stomach and pancreatic cancer; 1 point for lung, lymphoma, gynaecologic, bladder and testicular cancer); biomarkers (1 point each for platelet count ≥ 350 × 10^9^/L, haemoglobin < 100 g/L and/or use of erythropoiesis stimulating agents, white cell count ≥ 350 × 10^9^/L); and obesity (1 point for body mass index (BMI) ≥ 35 kg/m^2^). The PROTECHT, CONKO and CATS scores are modifications of the KS [23,24,25]. The PROTECHT score includes all KS parameters and adds gemcitabine and platinum-based chemotherapy (1 point each). The CONKO score replaces BMI with Eastern Cooperative Oncology Group Performance Status (ECOG PS) ≥ 2 (1 point). The CATS score [23] adds d-dimer and soluble p-selectin (1 point each) to KS. The CATS/MICA score includes only tumour type and d-dimer [26]. All the current models are designed for a baseline assessment only, overlooking the dynamic changes of an individual patients risk over time because of additional contributions of disease biology and anticancer therapies to TE risk [27,28].

Longitudinal characterisation of biomarker profiles, throughout the disease course and during treatment phases, may better predict the magnitude, timing, duration and clinical impact of haemostatic dysfunction and hence the opportunity for more applicable and balanced targeted preventative strategies. This study extensively profiled patients and developed a simple TE risk prediction model, using clinical parameters and readily available thrombogenic biomarkers, to allow real-time and dynamic risk stratification, which can direct personalised decision-making for preventative therapy.

## 2. Results

### 2.1. Patient Characteristics

From June 2014 to March 2016, 129 (76%) of 170 patients screened were included in Biomarkers of Thromboembolism in Lung cancer (BIOTEL) cohort. Patients excluded during the screening phase had established indications for therapeutic anticoagulation, TE prior to commencing anticancer therapy (*n* = 24, 14%), cardiovascular disease (*n* = 16, 9%), and 1 patient withdrew consent prior to baseline blood testing. Median follow-up for clinical events and survival was 22.0 months (range 5.6—31.3). Anticancer treatments included surgery (*n* = 12), CRT (47), CHT (36), and RT (34). During follow-up 52 (40%) patients received subsequent lines of anticancer therapy (median 2, range 2–4). For the purposes of the TE risk model derivation, only patients treated with chemotherapy and/or radiotherapy (*n* = 117) were included.

Because the TE risk appears somewhat unique according to different tumour cohorts, to test the scalability of our risk score, a further cohort (Biomarkers of Thromboembolism in Gastrointestinal Cancer, BIOTEGIC) was assessed. This included 71 patients commencing chemotherapy and/or radiotherapy for the treatment of gastrointestinal cancer from March 2012 to September 2015. Median follow-up for clinical events and survival in this cohort was 30.0 months (range 3.5–59.2). Patient characteristics for BIOTEL and BIOTEGIC are outlined in Table 1.

### 2.2. Biomarker Profiles

#### 2.2.1. Baseline

Biomarkers were assessed at sequential time-points, median 5 per patient (range 1–9). Elevation above normal reference limits for fibrinogen, d-dimer, and TEG-MA was observed in >75% of patients at baseline, concordant with a hypercoagulable profile. This was observed for patients with newly diagnosed NSCLC (fibrinogen ≥ 4 g/L (80%), TEG-MA ≥ 69 mm (74%), d-dimer ≥ 0.5 mg/L (93%) and recurrent NSCLC (fibrinogen ≥ 4 g/L (74%), TEG-MA ≥ 69 mm (74%), d-dimer ≥ 0.5 mg/L (86%).

#### 2.2.2. Longitudinal

**Figure 1:**
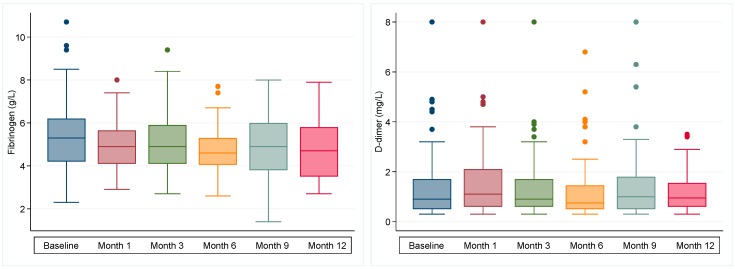
Longitudinal profile of fibrinogen and d-dimer among patients with NSCLC receiving chemotherapy and/or radiotherapy (*n* = 117). Box plots show median and interquartile range (IQR), with Tukey whiskers (±1.5*IQR).

Longitudinal biomarker profiles for fibrinogen and d-dimer are shown in Figure 1, with detailed summary statistics for these and other biomarkers including stratification by TE, disease progression, cancer stage, treatment type, treatment intent, and mortality, available as supplementary data (Table S1). A hypercoagulable profile persisted throughout the 12-month period in the majority of patients (75%). No patient received thromboprophylaxis during this time. Peak fibrinogen (median 5.3, IQR 4.2–6.2) and TEG-MA (median 72, IQR 69–76) were observed at baseline, peak d-dimer was observed at month one (median 1.1, IQR 0.6–2.1). Trough levels for these biomarkers were observed by month 6. Haemoglobin, white cell count and platelet count reduced over time with trough levels observed by month 3 and normalisation of peripheral blood parameters beyond month 6.

Although the overall biomarker profile did change over time, concordant with proposed concept that TE risk is dynamic, there was no discriminatory longitudinal pattern, nor absolute or relative change in biomarker value that reliably predicted for TE events or adverse outcome. The most potent risk prediction was determined by the absolute level at strategic time points—in particular, baseline and month one after commencement of anticancer therapy.

### 2.3. Thrombotic Events

Notably 14% (24/170) of screened patients experienced TE event prior to commencement of therapy (and screening)—and as such were excluded from the prospective cohort and analysis. Excluded patients were otherwise similar to the prospective cohort; 16/24 had advanced or metastatic disease, 17/24 adenocarcinoma histology and median age was 65 (range 36–76). The majority of TE events were PE (14/24, 64%). Importantly approximately half of these events occurred while waiting to commence anticancer therapy or during recent previous lines of cancer treatment and as such, potentially preventable.

Within the prospective study cohort BIOTEL, 17/117 (15%) patients experienced TE within six months of initiating anticancer treatment (median time to TE 48 days, range 1–151)—including 13 (11%) venous thromboembolism (VTE) [10 pulmonary embolism (PE), 4 deep vein thrombosis (DVT)] and 4 (3%) arterial TE (ATE). One event (PE) occurred during radiotherapy compared with 16/83 (19%) during chemotherapy. Median age at time of TE was 70 years (range 44–84). Of those who received chemotherapy, platinum-based was utilised in 93%, with emergent TE in those patients who received carboplatin (13/55, 24%), cisplatin (2/22, 9%), and paclitaxel (1/37, 3%).

Biomarker profiles of TE-positive patients were characterised by a progressive increase in d-dimer levels over time, with median d-dimer (mg/L) for TE-positive vs. TE-negative patients, at baseline (1.1 vs. 0.9, *p* = 0.27), 1-month (2.3 vs. 1.0, *p* = 0.01), and 3-month (4.8 vs. 0.9, *p* = 0.08), respectively. D-dimer elevation ≥ 1.5 mg/L (75th percentile) was observed at baseline in 29% of TE-positive and in a further 13% of TE-positive patients during treatment at 1-month.

### 2.4. Progression Free and Overall Survival

**Figure 2:**
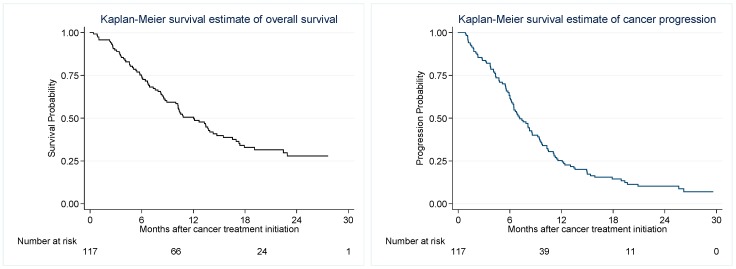
Kaplan Meier estimates of Overall Survival (OS) and Progression Free Survival (PFS).

Median follow-up for survival outcomes was 22.0 (range 5.6–31.3) months. During follow-up 81/129 (63%) patients died with median OS 12.7 months (range 1.1–31.3) and PFS 7.1 months (range 0.8–29.6), Figure 2. There was no difference in survival among patients with vs without TE (mortality HR 1.4, *p* = 0.24).

Patients with early progression and mortality (within six months) also demonstrated a baseline and sustained hypercoagulable state—reflected principally by elevated fibrinogen, d-dimer, and platelet count. In stage-adjusted analyses, pre-treatment biomarkers which predicted disease progression included fibrinogen ≥ 6 g/L (HR 2.3, 95% CI 1.2–4.3, *p* = 0.01), d-dimer ≥ 1.5 mg/L (HR 2.7, 95% CI 1.3–5.4, *p* = 0.01), white cell count ≥ 11 (HR 2.5, 95% CI 1.3–4.8, *p* < 0.01), and NLR ≥ 5 (HR 2.6, 95% CI 1.3–4.9, *p* < 0.01). Similarly, pre-treatment biomarkers which predicted mortality were fibrinogen ≥ 6 g/L (HR 2.3, 95% CI 1.1–4.8, *p* = 0.02), platelet count ≥ 350 × 109/L (HR 2.3, 95% CI 1.1–4.8, *p* = 0.02), white cell count ≥ 11 (HR 3.8, 95% CI 1.8–8.0, *p* < 0.01) and NLR ≥ 5 (HR 2.4, 95% CI 1.2–5.0, *p* = 0.01).

### 2.5. Risk Model Development

Associations of clinical parameters and thrombogenic biomarkers with TE incidence are outlined in Table 2 (expanded biomarkers in supplementary data; Table S2). Specifically, TE risk among patients receiving chemotherapy was comparatively higher than single modality radiotherapy (sHR 6.7, 95% CI 0.8–53.3, *p* = 0.07), with just one TE event observed in the smaller radiotherapy subgroup (1/34, 3%). Other than chemotherapy, performance status was the only other statistically significant clinical predictor of TE, with stage-adjusted sHR for TE (ECOG PS ≥ 2 vs. <2) for all patients 5.9 (95% CI 0.8–41.8, *p* = 0.08) and chemotherapy patients 8.6 (95% CI 1.2–63.0, *p* = 0.03). Stage, histology, disease status (new vs. recurrent NSCLC), age, sex, comorbidity, and prognosis (early mortality and early progression) did not demonstrate statistically significant associations with emergent TE events, Table 2.

D-dimer proved the most potent individual biomarker and had the greatest TE predictive capacity with repeated assessments, Table 2. Stage-adjusted sHR for TE (d-dimer ≥ 1.5 mg/L vs. <1.5 mg/L, at either baseline or 1-month) for all patients was 2.6 (95% CI 0.8–7.7, *p* = 0.10) and chemotherapy patients 3.1 (95% CI 1.0–9.8, *p* = 0.05). Of patients with d-dimer <1.5 mg/L at both baseline and 1-month (68/117, 58%), only 6 patients developed TE, all of whom had baseline fibrinogen ≥ 4 g/L and d-dimer ≥ 0.5 mg/L. Among patients with d-dimer <1.5 mg/L, TE occurred more commonly with baseline fibrinogen ≥ 4 g/L compared to <4 g/L: entire cohort 10% vs. 0% (*p* = 0.16) and chemotherapy cohort 16% vs. 0% (*p* = 0.13). TE occurred more commonly with baseline d-dimer ≥ 0.5 mg/L compared to <0.5 mg/L: entire cohort 12% vs. 0% (*p* = 0.11) and 19% vs. 0% (*p* = 0.05) chemotherapy cohort. Correspondingly, TE occurred more commonly with both baseline fibrinogen ≥ 4 g/L and d-dimer ≥ 0.5 mg/L compared to either baseline fibrinogen <4 g/L or d-dimer <0.5 mg/L: entire cohort 15% vs. 0% (*p* = 0.04) and 23% vs. 0% (*p* = 0.02) chemotherapy cohort.

Of the novel biomarkers in the expanded panel, only thrombin generation (endogenous thrombin potential (ETP): absolute, ETP Peak, and Velocity Index), alpha-2-macroglobulin and thrombin-antithrombin (TAT) were associated with TE (Supplementary Data Table S2). These data were not included in risk modelling due to limited sampling and are discussed in further detail in a separate publication.

Variables associated with TE in stage-adjusted analyses (Table 2), were assessed in a multivariable model: chemotherapy treatment, ECOG PS ≥ 2, age ≥ 70, NLR ≥ 5, and the combined d-dimer/fibrinogen covariate (baseline fibrinogen ≥ 4 g/L AND d-dimer ≥ 0.5 mg/L; OR d-dimer ≥ 1.5 mg/L at either baseline or month 1). Only chemotherapy (*p* = 0.03) and the combination d-dimer/fibrinogen covariate (*p* < 0.01) retained a significant association with TE. The final model (model 1) included the following TE risk factors: (i) chemotherapy treatment, (ii) baseline d-dimer ≥ 1.5 mg/L, (iii) month one d-dimer ≥ 1.5 mg/L, and (iv) baseline fibrinogen ≥ 4 g/L and d-dimer ≥ 0.5 mg/L. Exploratory models were created with model 2 restricted to baseline variables [(i) and {(ii) or (iii)}] and model 3 including performance status [(i) and {(ii) or (iii) or (iv)}] and ECOG PS ≥ 2). Variables and risk assessment algorithms for derived and previously published models are summarised in Table 3.

### 2.6. Risk and Rates of TE According to Published Risk Models

All derived models demonstrated greater potency with regards to selection of high TE risk, compared to published models (range 59–72% vs. 24–64%; Table 4). Model 1 classified 72% of patients as high TE risk, with a TE incidence of 27%, compared to 0% among those classified as low risk. Importantly, while only available for a subset of the cohort, thrombin generation and low risk stratified patients was concordant, supporting biologic validity of the risk stratification.

Within this cohort, KS, PROTECHT, and CONKO scores classified high TE risk in 24%, 64%, and 48% patients, with measured TE rates of 20%, 21%, and 25% respectively. More importantly, among those classified as “low risk”, TE rates were high with 19%, 16% and 14% for KS, PROTECHT and CONKO respectively.

**Figure 3:**
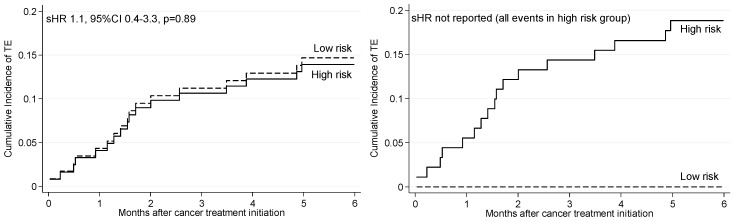
Nelson-Aalen cumulative hazard function for risk of thromboembolism. (**A**) Study derived TE risk; (**B**) Khorana TE risk model.

The sHR for TE (high vs. low risk) for each of the models is presented in Table 4. For model 1, no TE events occurring in the low risk group and with regards to TE prediction, among all patients number needed to treat (NNT) was 5.29, reducing to 3.75 considering only those receiving chemotherapy. For model 2 (baseline variables) and model 3 (including ECOG PS), sHR for TE were 8.2 (95% CI 1.1–61.6) and 12.3 (95% CI 1.7–91.8), respectively. More importantly, KS, PROTECHT and CONKO scores did not predict TE. All published models had lower BIC, higher c-index, and higher sensitivity. Cumulative incidence functions for TE according to study-derived and KS TE risk model status are presented in Figure 3. PROTECHT and CONKO models are shown in supplementary data (Figure S1).

Within BIOTEGIC 7/71 (10%) patients experienced TE, the developed model did indeed demonstrate equipotency with 71% sensitivity and 39% specificity. The model identified 62% of patients with gastrointestinal cancer as high TE risk. Comparatively, the KS, PROTECHT and CONKO scores each predicted only one of seven TE events (sensitivity 14%). Assessments of TE risk for high vs. low risk patients were non-significant due to small cohort size (*n* = 71) and number of TE events (*n* = 7). 

### 2.7. Progression and Mortality According to TE Risk Classification 

Derived TE risk models 1 and 3 also predicted for death and cancer progression (Table 3). Kaplan Meier estimates for OS and PFS according to TE risk model status are presented as supplementary data (Figure S1).

## 3. Discussion

This prospective observational and longitudinal study demonstrated a high TE rate among patients with NSCLC (both newly diagnosed and at relapse), prior to commencing any therapy (14%) and during the first 3–6 months of therapy (16%), justifying the need for early identification and intervention with targeted P-TP. The data proposes the utilization of simple predictive markers, which have been previously investigated for TE risk assessment; however, this study extends beyond prior published data, combining a more extensive panel of parameters and repeated assessment to capture the dynamic TE risk profile and optimize test sensitivity.

Biomarker profiles depicted a procoagulant state for patients newly diagnosed (≥78%) and those with disease recurrence (≥74%). Although this profile persisted over the observed 12-month period, there was notable amplification during the first 3–6 months of anticancer treatment, when the majority (79%) of TE occurred. This is concordant with published data [6,7,31] and randomised studies implementing P-TP during this period, which demonstrated excess TE events in control groups [9,10,11,12]. This represents a critical time period—both for delivery of anticancer therapy and an opportunity to proactively intervene with TE preventative strategies—noting that the prothrombotic profile also correlated with poorer outcomes in terms of both early disease progression and reduced overall survival.

P-TP is a proven and safe preventative strategy, with up to 80% reduction in cancer associated TE in studies [16]. However, the perceived heterogeneity in CAT risk reflected by the variation in reported incidence [8,32,33], as well as concerns regarding excess cumulative bleeding risk, has restricted appropriate “routine” P-TP utilisation among ambulatory patients—where the majority of patients are treated and approximately 80% of cancer associated TE events occur [5,21]. Heterogonous and dynamic TE risk (incidence ranging 3–40% across studies) [8,32,33] confers clinical uncertainty and misconception of risks—which further contributes to the global under utilisation of appropriate P-TP. Impacting further is the focus on an “absence” of a statistically definitive survival benefit in many of the intervention studies [9,34]. Although improved survival remains important, benefits of prevention extend well beyond this, given an index TE event confers not only reduced survival, but substantial morbidity with a significant impact on quality of life and delivery of effective anticancer therapy [1,2,3,4]. Oral anticoagulants are now being trialed as TE preventative strategies among patients with cancer in an ambulatory setting. Recently published data and data presented at an international meeting, demonstrated a benefit in Khorana risk score patients ≥2 with broad representation of histologies [35,36]. VTE was reduced from 10.2% in the placebo arm compared to 4.2% using apixaban 2.5 mg twice daily for 180 days (HR 0.41, *p* < 0.001) [35]. Results for rivaroxaban were less convincing with VTE reduction from 8.8% in the placebo arm compared to 6.0% using rivaroxaban 10 mg daily for 180 days (HR 0.66, *p* = 0.10) [36]. While promising, neither trial compared efficacy against current standard of care (low molecular weight heparin), and utilization of the Khorana score as demonstrated in this and other validation cohorts, likely limits the number of patients achieving benefit while leaving other at-risk patients untreated.

Clinicians are seeking simple and targeted guidance, for application in patients who are at high risk of TE and time periods, avoiding unnecessary intervention when TE risk is low. Development of a robust predictive tool with decision-making algorithm, both available and easy to use real-time in the clinic, will enable this personalised approach when overarching management decisions are being made.

In recognition of the need to target P-TP, guidelines including from the American Society of Clinical Oncology (ASCO) and European Society of Medical Oncology (ESMO), acknowledge the use of prior TE risk assessment tools [18,19]. However, with existing models, specificity was prioritised over sensitivity and conservative biomarker thresholds were applied. With an inability to appropriately direct preventative therapies, the 2016 updated National Comprehensive Cancer Network (NCCN) guidelines removed previous recommendations to consider KS [20].

Reflective of previous findings, existing TE models [22,24,25] demonstrated low predictive capacity within the study cohort and the derived model revealed a markedly improved sensitivity (up to 75% increase) and efficiency (lowest BIC). A defining feature of the study model is the further reassessment at month one, allowing identification of patients with amplified thrombogenic biomarkers (and hence increased TE risk) after commencing anticancer treatment, otherwise overlooked by baseline models. Omitting reassessment reduced model performance (model 2, Table 4), though still better than comparative baseline models. Adding ECOG PS assessment like the CONKO study in pancreatic cancer [25], fewer patients were classified as high TE risk resulting in the greatest specificity, PPV, and TE risk estimate (model 3, Table 4). However, subjectivity of ECOG PS and relative reduction in sensitivity, model 1 remained the preferred model to guide risk-directed primary prevention.

Khorana, CATS, PROTECHT and CONKO risk models used pre-treatment biomarkers only [22,23,24,25]. While predicting TE in mixed cancer cohorts, stratification is largely based on cancer type, and as demonstrated within BIOTEL and BIOTEGIC, these models demonstrate low predictive capacity within specific cancer cohorts [27,28]. None of the biomarkers included in KS (platelet count, white cell count, and haemoglobin) predicted TE in our cohorts and BMI was irrelevant as no patient reached the 35 kg/m^2^ threshold, as demonstrated previously within the CONKO pancreatic cancer cohort [25]. As 93% of patients received guideline appropriate platinum-based chemotherapy [37], the PROTECHT score adding 1 point for platinum (and gemcitabine) chemotherapy, did not add to discriminative ability. Poor performance status was common (74% of patients had ECOG PS ≥ 2), and as with the CONKO pancreatic cohort [25], predicted TE in this NSCLC cohort. The CATS model [23], weighs heavily on P-selectin, which is an investigational biomarker and not routinely available in diagnostic laboratories. Consequently, the CATS model has not undergone external validation or use in routine clinical practice. The extended panel of investigational thrombogenic biomarkers in this study, added minimal differentiation with regards to TE risk prediction and has largely been used for biological rationale.

In recent works published after completion of the current study, a new TE risk model was derived from updated CATS cohort data, with subsequent validation on an independent prospective cohort (the MICA cohort) [26]. Only two of the original CATS model variables (tumour type and d-dimer) were included in the derived risk model. Thus when the CATS/MICA model is applied to a single tumour group, TE risk stratification relies solely on baseline d-dimer levels. We have shown to be commonly elevated among lung cancer patients with and without TE, not excessively elevated in those who subsequently develop TE, and that elevation can occur after commencement of anticancer therapy (i.e., not detected on baseline screening). In this lung cancer cohort, baseline d-dimer (as a continuous variable) was not predictive of TE (Table 2), suggesting limited ability of the proposed risk model to stratify the cohort according to TE risk. Using the CATS/MICA nomogram model to identify patients at high TE risk (defined as >10% cumulative TE incidence), the required risk score is approximately 110. This would include approximately 50 points for lung or gastrointestinal cancer diagnosis (high risk tumours) and therefore, at least 60 points for d-dimer, requiring markedly elevated d-dimer (>7 ug/mL). Concordantly to identify ‘high TE risk’ defined as cumulative TE incidence 27%, (CATS/MICA score of approximately 190), a d-dimer score of 140 (d-dimer of >32 ug/mL) would be required, which is not only clinically improbable but above the limits of the nomogram itself.

Concordant with other studies, our BIOTEL cohort also defined important time periods of high TE risk—recognising the first 3–6 months as the highest risk period justifying application of targeted P-TP [6,7,9,10,11,12]. Meta-analysis of randomised trials of P-TP during chemotherapy have confirmed the effectiveness of P-TP in this setting, but in the absence of risk-stratification, an unacceptable high number needed to treat (NNT = 60) [16]. The application of risk scores, such as with KS, has suggested improvement (NNT = 15–25 for KS ≥ 3 applied to the PROTECHT and SAVE-ONCO studies), but with a low sensitivity [38]. In this cohort and others, a large proportion of TE events occurred among patients designated with a low risk score (KS < 3) [23,39]. Our proposed model does demonstrate greater potency, considering both risk status and duration. Based on the stratification algorithm, high TE risk patients would receive P-TP for a minimum of three months, or until cessation of chemotherapy, to a maximum of six months. Given the substantial number of events occurring between diagnosis and commencement of anticancer therapy, we advocate for early risk assessment and early initiation of primary prevention for those classified as high TE risk. Given NNT of 4 in this NSCLC cohort and 9 in BIOTEGIC cohort, benefits of risk-adapted therapy would be improved by up to 3-fold with the study derived risk model. This is being tested in the actively accruing TARGET-TP trial (ACTRN12618000811202) of enoxaparin P-TP versus no P-TP for high TE risk patients with lung or gastrointestinal cancer.

We acknowledge several limitations of this study, in particular patient population size and single centre recruitment. However, importantly, the model derivation cohort included a real-world NSCLC population with complex and comorbid patients often excluded from interventional clinical studies. Moreover, despite smaller study populations and consequent wide confidence intervals, point estimates for TE risk evaluation are high (up to 12-fold for high versus low risk).

## 4. Materials and Methods

### 4.1. Biomarker Selection

While extensively investigated, no currently recognised individual biomarker adequately identifies individual patient risk. Many proposed biomarkers are still considered investigational and only available in highly specialised laboratories. This biomarker panel was selected to further explore the pathophysiology of cancer associated TE with the aim to identify more sensitive and specific biomarkers that could be utilised real-time in routine diagnostic laboratories. The panel included markers of coagulation activation, thrombin generation and fibrinolysis, as well as a global assessment of the haemostatic system (thromboelastography—TEG). Preliminary data has demonstrated correlation of TEG with in vivo markers of coagulation activation and fibrinolysis and peri-operative TE risk, but only limited data in cancer-associated hypercoagulable states.

### 4.2. Cohort Selection

This study was undertaken in patients with lung cancer representing the most commonly diagnosed and deadly cancer globally, with significant incidence of thromboembolic complications. While recognizing tumour group heterogeneity, scalability of the risk score was tested in a second cohort comprising gastrointestinal cancers, selected for diagnostic frequency, TE incidence and high ambulatory care chemotherapy exposure (neoadjuvant, adjuvant, palliative and definitive settings).

The BIOTEL cohort (Biomarkers of Thromboembolism in Lung cancer) is a single centre prospective cohort study, which longitudinally profiled patients with NSCLC referred for anticancer therapy from July 2014 to March 2016, for both clinical events as well as thrombogenic biomarkers. Patients were enrolled at diagnosis or relapse, with treatment strategies at the discretion of the treating clinician, and excluded only if they were receiving anticoagulation for any indication. Patients provided written informed consent to participate in the studies, which were approved by the institutional ethics committee (ethics no. 14/31 approved 16 June 2014 and 12/37 approved 07 May 2012). The derived model from BIOTEL was then tested on an independent cohort of patients with gastrointestinal cancer from the BIOTEGIC cohort (Biomarkers of Thromboembolism in Gastrointestinal Cancer), to assess applicability across another tumour cohort. BIOTEGIC replicated BIOTEL in terms of data collection, blood samples and endpoints. 

### 4.3. Data Collection

Sequential clinical and laboratory parameters were collected at baseline, week 1, months 1, 3, 6, 9 and 12 following commencement of anticancer therapy. Follow-up for all patients continued 3-monthly until 12 months after enrolment of the last patient (March 2017). Variables included: demographics; performance status; smoking history; cancer stage, NSCLC histology and mutation status; treatment regimens; body mass index (BMI); history of TE; medical comorbidities and combined comorbidity risk scores of Colinet and Charlson [40,41]. Thrombogenic biomarkers included: thromboelastography (TEG-R, TEG-K, TEG-Angle, and TEG-MA), fibrinogen, d-dimer, platelet count, haemoglobin, white cell count and differential, with calculated neutrophil to lymphocyte ratio (NLR) and platelet to lymphocyte ratio (PLR). Additional biomarkers tested on a smaller subset of patients included endogenous thrombin potential (ETP-absolute, peak, and velocity index), alpha-2-macroglobulin, von Willebrand factor antigen (vWF-Ag), factor VIIIc, fibrin monomers (FM), thrombin-antithrombin (TAT) and phospholipids (PPL).

### 4.4. Blood Samples 

Venous blood samples were collected in standard vacutainers containing trisodium citrate solution at 0.106 mol/L and EDTA. Samples were analysed real time in the diagnostic laboratory. Full blood count parameters using automated analyser, activated partial thromboplastin time, prothrombin time, fibrinogen, d-dimer, vWF-Ag, FVIIIc using STA^®^-Liatest^®^, phospholipids using Stago STA automated platforms (Stago Group, Asnieres sur Siene Cedex, France), TEG using Haemonetics TEG 5000 Thrombelastograph Hemostasis Analyzer (Haemonetics, MA, USA), TAT and FM using enzyme-linked immunosorbent assay (ELISA). Where blood samples were not obtainable (patient refusal or transfer of care to a different health centre), clinical follow-up data continued to be collected.

### 4.5. Outcomes

The primary outcome measure of BIOTEL was objectively confirmed arterial TE (ATE) or venous TE (VTE) within six months of treatment initiation; bleeding events (clinically relevant minor, major and fatal). Secondary outcomes were disease response (RECIST criteria), overall survival (OS) and progression free survival (PFS). For real world relevance and pragmatic reasons, screening imaging for TE beyond standard of clinical care was not prescribed. TE events included in this study were confirmed events using objective imaging methods (ultrasound or computed tomography) or incidental TE detected on radiological surveillance. Deaths were reviewed for possible attribution to TE and all deaths within 30 days of any TE were reported. The rationale for including both venous and arterial events, recognizes not only the clinical importance of both but also the emergent risk profiles and broader mechanisms of action, with the addition of targeted therapy. 

### 4.6. Sample Size

We aimed to recruit 120 participants, with expected TE rate of 5–15% enabling estimation of the true TE incidence rate with reasonable precision as indicated by the 95% confidence intervals (95% CI) for the following TE event rates: 5% (95% CI 2–11%), 10% (95% CI 5–17%) and 15% (95% CI 9–23%). Participants had biomarker assessments at a minimum of 7 time points (see data collection) providing multilevel analysis of 9240 data points for all biomarkers, enabling an estimation of important summary features of the biomarker curve over time and treatment course.

### 4.7. Statistical Analysis

The incidence of TE was estimated from the date of anticancer treatment initiation until 6 months. Death was regarded as a competing risk and living patients without TE were censored at 6 months or last study follow-up. The association between variables (clinical parameters and thrombogenic biomarkers) and TE occurrence was assessed using stage-adjusted and multivariable Fine and Gray competing risk proportional hazards regression models with results reported as sub-distribution hazard ratio (sHR). Corresponding cumulative incidence functions were obtained from the same models. Competing risk assessment is especially important in lung cancer cohorts where patients are at risk of both death and TE and with expected high early mortality rates, the absence of a competing risk model can lead to overestimation of event rates [42].

Thrombogenic biomarker levels were explored as continuous variables and as binary variables using (i) standard reference ranges, (ii) pre-defined prediction thresholds (platelet count >350 × 10^9^/L, haemoglobin < 100 g/L, white cell count >11 × 10^9^/L, d-dimer >1.44 mg/L, neutrophil-lymphocyte ratio (NLR) ≥ 2.5 and NLR ≥ 5.0, platelet-lymphocyte ratio (PLR) ≥ 150 and PLR ≥ 300 [22,23,43,44], and (iii) at thresholds informed by the data (i.e., median, 25th and 75th percentiles).

Variables associated with TE in univariable analyses were assessed in multivariable with those retaining a significant association (*p* ≤ 0.05) included in our final model. Additional exploratory models were developed using (i) baseline variables, to allow comparison with existing risk models [22,23,24,25], and (ii) incorporating performance status [25]. Derived models were compared to published models Khorana, CATS, PROTECHT, and CONKO [22,23,24,25], using Bayesian Information Criterion (BIC) for the competing risk model and cause-specific assessments of the C-index (area under receiver operating characteristic (ROC) curve), and the classification measures: sensitivity, specificity, positive predictive value (PPV), negative predictive value (NPV). The best performing model was selected based on efficiency (lowest BIC) and ability to achieve high sensitivity. While recognising the importance of specificity, sensitivity was prioritised given the serious adverse outcomes of TE and availability of proven preventative therapies. For Khorana, PROTECHT and CONKO models, high risk was defined as a score ≥ 3 and low risk as a score <3 [22,24,25]. As the CATS model could not be applied to the cohort (soluble p-selectin not available), performance measures were taken from the literature [23].

The Kaplan-Meier method was used to estimate PFS and OS among groups defined by TE risk classification. OS was defined as time to death by any cause (living patients censored at last follow-up), and PFS was defined as time to first medical documentation of cancer progression (RECIST criteria) [45], or death (patients in remission or with stable disease censored at last follow-up). Associations between our modelled TE risk status and OS and PFS were analysed using stage-adjusted Cox proportional hazards regression with results reported as hazard ratio (HR).

All statistical analyses were performed using Stata v14.0 [46]. All tests were two-sided using a 5% significance level and corresponding 95% confidence intervals (CI) calculated.

## 5. Conclusions

This derived TE risk assessment tool provides pragmatic, real-time risk stratification and personalised decision-making with regards to appropriate P-TP. Moreover, this model can be used at any stage during the patient’s disease and/or treatment journey. Concordant with the newly revised model from the CATS/MICA cohorts we identified the significant role of d-dimer as a predictive biomarker, but with greater potency and discrimination within tumour groups by using a defined threshold and with the addition of fibrinogen. We acknowledge that the limitations of this study included a smaller patient populations and single centre recruitment. However, both BIOTEL and BIOTEGIC cohorts included real-world lung and gastrointestinal cancer populations with complex and comorbid patients often excluded from interventional clinical studies. Equally, despite the smaller study populations and consequent wide confidence intervals, point estimates for TE risk evaluation were substantial, with up to 12-fold increased risk for high vs. low risk.

Most importantly, the prediction tool is simple, using readily available biomarkers, allowing real-time and sequential TE risk assessment in the clinic, without barrier to implementation. The model is both predictive and reactive, providing a personalised, risk-targeted algorithm for P-TP. This risk-score based decision-making algorithm is currently being tested in a randomised interventional study (TARGET-TP), among patients with both NSCLC and gastrointestinal cancer receiving anticancer therapy. Their risk profile is established prior to the commencement of anticancer therapy and designated high TE risk patients are randomized to preventative dose low molecular weight heparin for a minimum of 3 months (or to completion of therapy or maximum of 6 months) or no thromboprophylaxis. All low TE risk patients continue on study within the observational arm. Importantly patients are re-profiled at 1 month and can enter randomisation at that point if identified at high TE risk. This strategy mirrors the findings in BIOTEL and allows patients to have a dynamic risk assessment and not be overlooked if the TE risk emerges after the commencement of therapy. We look forward to sharing these results at the completion of the study follow-up. We also hope to consider a step-wedged design going forward and include subsequent tumour cohorts with a similar risk and preventative strategy.

## Figures and Tables

**Table 1:** Patient pre-treatment characteristics.

Characteristic	BIOTEL (*n* = 117)	BIOTEGIC (*n* = 71)
Patient characteristic		
Male sex, n (%)	74 (63)	42 (59)
Median age (range), years	67 (44—89)	63 (31–90)
Stage I-IIIA, n (%)	57 (49)	43 (61)
Stage IV, n (%)	60 (51)	28 (39)
ECOG performance status ≥ 2, n (%)	86 (74)	10 (14)
Previous thromboembolism, n (%)	9 (8)	2 (3)
Primary treatment, n (%)		
Chemoradiotherapy	47 (36)	50 (70)
Chemotherapy	36 (28)	18 (25)
Radiotherapy	34 (26)	3 (4)
Curative	15 (44)	3 (100)
Palliative	(56)	0 (0)

**Table 2:** Associations between biomarkers and clinical parameters with thromboembolism, by stage-adjusted Fine and Gray competing-risks regression in the NSCLC cohort.

Risk Factor	All (*n* = 117)			Chemotherapy (*n* = 83)		
	sHR ^1^	95% CI	*p*	sHR^1^	95% CI	*p*
Baseline Biomarkers						
D-dimer, continuous	1.8	0.7–4.7	0.23	2.1	0.8–5.6	0.14
D-dimer ≥ 0.5 mg/L	All events in high risk group			All events in high risk group		
D-dimer ≥ 1.5 mg/L	1.6	0.6–4.4	0.38	2.1	0.8–5.8	0.16
Fibrinogen ≥ 4.0 g/L	1.2	0.3–4.3	0.80	1.4	0.4–5.5	0.60
Platelet count ≥ 350 × 10^9^/L	1.2	0.5–3.3	0.69	1.5	0.6–4.1	0.40
Haemoglobin < 100 g/L	0.7	0.1–4.6	0.70	1.2	0.2–7.3	0.84
White cell count ≥ 11.0 × 10^9^/L	0.9	0.3–2.4	0.82	0.9	0.3–2.5	0.86
NLR ≥ 5.0	0.4	0.1–1.1	0.07	0.4	0.1–1.2	0.11
PLR ≥ 300	0.7	0.2–2.5	0.59	0.9	0.3–2.9	0.90
TEG-MA ≥ 76 mm	0.8	0.3–2.4	0.68	1.2	0.4–3.7	0.71
TEG-Angle ≥ 77 degrees	1.7	0.6–4.7	0.34	1.7	0.6–5.0	0.34
TEG-R ≤4.5 min	1.8	0.7–4.9	0.20	1.3	0.5–3.7	0.60
TEG-K ≤0.9 min	1.5	0.5–4.4	0.47	1.5	0.5–4.8	0.46
Month 1 Biomarkers						
D-dimer ≥ 1.5 mg/L	1.6	0.6–4.4	0.33	1.6	0.6–4.6	0.35
Fibrinogen ≥ 6.0 g/L	1.5	0.6–4.3	0.40	2.3	0.8–6.6	0.11
Platelet count ≥ 350 × 10^9^/L	1.7	0.6–4.7	0.27	2.3	0.8–6.5	0.10
Combined Biomarkers						
Baseline d-dimer ≥ 0.5 mg/L and fibrinogen ≥ 4.0 g/L	2.1	0.5–7.8	0.29	2.7	0.7–10.8	0.16
Baseline d-dimer ≥ 1.5 mg/L or month 1 d-dimer ≥ 1.5 mg/L	2.6	0.8–7.7	0.10	3.1	1.0–9.8	0.05
TEG-R ≤4.5 min and TEG-K ≤0.9 min	2.8	0.9–8.7	0.08	1.8	0.5–6.0	0.34
Baseline d-dimer ≥ 0.5 mg/L and fibrinogen ≥ 4.0 g/L, or baseline d-dimer ≥ 1.5 mg/L	6.1	0.8–49.0	0.09	8.5	1.0–71.4	0.05
Baseline d-dimer ≥ 0.5 mg/L and fibrinogen ≥ 4.0 g/L, or baseline d-dimer ≥ 1.5 mg/L, or month 1 d-dimer ≥ 1.5 mg/L	All events in high risk group			All events in high risk group		
Clinical Factors						
Treatment factors						
Chemo (CRT/CHT) vs. RT	6.7	0.8–53.3	0.07	Not applicable		
Disease factors						
Stage IV (vs. I-III)	1.1	0.3–4.2	0.83	1.1	0.3–4.2	0.85
Stage IIIB/IV (vs. I-IIIA)	1.7	0.6–5.0	0.30	1.2	0.4–3.5	0.71
New (vs. recurrent)	2.4	0.5–10.2	0.25	1.5	0.3–6.3	0.25
Adenocarcinoma (vs. other)	1.3	0.5–3.2	0.62	1.3	0.5–3.8	0.58
Patient factors						
Age ≥ 70 years	2.6	1.0–7.0	0.06	0.4	0.2–1.2	0.11
Female sex	1.5	0.6–3.8	0.43	1.4	0.5–4.0	0.49
BMI>30 kg/m^2^ ^2^	0.7	0.2–3.2	0.66	0.5	0.1–4.2	0.52
ECOG PS ≥ 2	5.9	0.8–41.9	0.08	8.6	1.2–63.0	0.03
Ever smoker	2.0	0.3–15.9	0.50	1.7	0.2–12.9	0.63
Colinet score >9	1.7	0.4–4.0	0.68	1.2	0.4–3.8	0.74
Charlson score >3	2.2	0.8–6.1	0.12	1.9	0.7–5.1	0.22
Prognosis						
Progression ≤6 months	1.1	0.4–2.9	0.91	1.4	0.6–3.5	0.48
Death ≤6 months	1.2	0.5–3.1	0.64	1.4	0.5–4.1	0.59

^1^ Stage-adjusted (stage IIIB/IV vs. stage I-IIIA) sub-distribution hazard ratio; ^2^ BMI assessed at >30 kg/ m^2^ as no patients in cohort had BMI>35 mg/m^2^, threshold applied in existing TE risk scores. TE, thromboembolism; PD progressive disease; OS, overall survival; HR, hazard ratio; B; baseline (prior to initiation of anticancer treatment); M1, one month from initiation anticancer treatment; NLR, neutrophil to lymphocyte ratio; PLR, platelet to lymphocyte ratio; CRT, chemoradiotherapy; CHT, chemotherapy; Chemo, chemoradiotherapy or chemotherapy; RT, radiotherapy; BMI, body mass index; ECOG PS, eastern cooperative oncology group performance status.

**Table 3:** Criteria for defining high risk of thromboembolism according to study derived and published risk models. All assessments occur at baseline unless otherwise specified.

Model	Risk Assessment	Score	High Risk
Study Derived	Fibrinogen ≥ 4.0 and D-dimer ≥ 0.5	1	Score ≥ 1
	D-dimer ≥ 1.5	1	
	D-dimer ≥ 1.5 month 1	1	
Khorana	Very high risk tumour site (stomach, pancreas)	2	Score ≥ 3 ^1^ or Score ≥ 2 ^2^
	High risk tumour site (lung, lymphoma, gynecologic, genitourinary excluding prostate)	1	
	Platelet >350 × 10^9^/L	1	
	Hb < 100 g/L or use of ESA	1	
	Leukocyte count > 11 × 10^9^/L	1	
	BMI ≥ 35 kg/m^2^	1	
CATS	As for Khorana score but add:		Score ≥ 3 ^3^
	Soluble P-selectin ≥ 53.1 ng/mL	1	
	D-dimer ≥ 1.44 ug/mL	1	
CONKO	As for Khorana score but remove BMI≥ 35 kg/m^2^ and add:		Score ≥ 3 ^3^
	ECOG PS ≥ 2	1	
PROTECHT	As for Khorana score and add:		Score ≥ 3 ^3^
	Gemcitabine chemotherapy	1	
	Platinum chemotherapy	1	
CATS/MICA ^5^	Tumour-site risk category (low or intermediate)	0	Score ≥ 110 ^4^
	Tumour-site risk category (high)	50	
	Tumour-site risk category (very high)	95	
	D-dimer concentration (0.1–0.5 ug/mL)	0–10	
	D-dimer concentration (0.5–2.0 ug/mL)	10–30	
	D-dimer concentration (2.0–8.0 ug/mL)	30–60	
	D-dimer concentration (>8.0 ug/mL)	>60	

^1^ High risk threshold originally proposed by Khorana et al. [22]. ^2^ High risk threshold utilized in the CASINI and AVERT clinical trials of primary thromboprophylaxis [29,30]. ^3^ High risk threshold applied for Khorana score derivatives as per original Khorana model. ^4^ High risk threshold defined as cumulative TE incidence of 10–15%, reflecting nomogram score of 110–140 [26]. ^5^ Points for CATS/MICA score approximated from published nomogram [26].

**Table 4:** Thromboembolism risk classification, estimated cumulative incidence of thromboembolism, and predictive ability of newly derived and established thromboembolism risk models in the NSCLC cohort.

Risk Model	Cohort	High Risk		Low Risk		Prediction of Thromboembolism							Prediction of Mortality	Prediction of Progression
		No. (%)	Cum. TE % ^a^	No. (%)	Cum. TE %^a^	Sensitivity (95% CI)	Specificity (95% CI)	PPV (95% CI)	NPV (95% CI)	sHR ^b^ (95% CI), *p*-Value	BIC ^c^	AUC ^d^ (95% CI)	HR ^e^ (95% CI), *p*-Value ^f^	HR ^e^ (95% CI), *p*-Value ^f^
Model 1 ^g,i^	CHT	60 (72)	26.5	23 (28)	0.0	100 (79–100)	34 (23–47)	27 (16–40)	100 (85–100)	All TE in high risk group	127	0.67 (0.61–0.73)	2.2 (1.2–4.2), 0.015	1.9 (1.2–3.2), *p* = 0.011
Model 1 ^g,i^	CHT/ RT	60 (51)	26.5	57 (49)	3.1	100 (81–100)	27 (19–37)	19 (11–29)	100 (87–100)	All TE in high risk group	NR ^e^	0.64 (0.59–0.68)	1.5 (0.9–2.3), 0.096	1.6 (1.1–2.4), *p* = 0.018
Model 2 ^g,j^	CHT	56 (67)	26.6	27 (33)	3.7	94 (70–100)	39 (27–52)	27 (16–40)	96 (81–100)	8.2 (1.1–61.6), *p* = 0.04	135	0.66 (0.58–0.75)	1.6 (0.9–2.9), 0.078	1.5 (0.9–2.4), *p* = 0.081
Model 3 ^g,k^	CHT	49 (59)	30.6	34 (41)	2.9	94 (70–100)	49 (37–62)	31 (18–45)	97 (85–100)	12.3 (1.7–91.8), *p* = 0.01	131	0.72 (0.62–0.80)	2.4 (1.4–4.2), 0.0001	2.2 (1.5–3.4), *p* = 0.002
Khorana ^g,l^	CHT	20 (24)	20.2	63 (76)	18.9	25 (7–52)	76 (64–86)	20 (6–44)	81 (69–90)	1.1 (0.4–3.3), *p* = 0.89	143	0.51 (0.39–0.63)	2.01 (1.2–3.4), 0.005	1.9 (1.2–3.2), *p* = 0.002
PROTECHT ^g,m^	CHT	53 (64)	20.9	30 (36)	16.3	69 (41–89)	37 (26–50)	21 (11–34)	83 (65–94)	1.3 (0.5–3.7), *p* = 0.60	142	0.53 (0.40–0.66)	1.8 (1.1–2.9), 0.013	1.9 (1.2–2.8), 0.002
CONKO ^g,n^	CHT	40 (48)	25.0	43 (52)	13.8	63 (35–85)	55 (43–67)	25 (13–41)	86 (72–95)	1.9 (0.7–5.3), *p* = 0.71	141	0.59 (0.45–0.73)	2.6 (1.7–4.2), <0.001	2.8 (1.9–4.2), <0.001
CATS ^h,o^	CHT	-	-	-	-	64 (NR)	82 (NR)	20 (NR)	97 (NR)	-	-	-	-	-

(a) Estimated TE incidence at six months from cumulative incidence function for TE with death as a competing risk; (b) Univariable sHR; (c) lowest BIC reflects most efficient model; (d) Area under ROC curve; (e) Stage-adjusted (stage IIIB/IV vs. stage I-IIIA) Cox proportional hazards regression; (f) p-value for mortality and progression among patients with high vs. low TE risk; (g) Model applied to NSCLC study population; (h) Model applied to mixed cancer population (published data); (i) Model 1: high risk if: (i) receiving chemotherapy and ((ii) baseline d-dimer ≥ 1.5 mg/L or (iii) month one d-dimer ≥ 1.5 mg/L or (iv) baseline fibrinogen ≥ 4 g/L and d-dimer ≥ 0.5 mg/L); (j) Model 2: high risk if: (i) and ((ii) or (iv)); (k) Model 3: high risk if: (i) and ((ii) or (iii) or (iv)) and ECOG PS ≥ 2; (l) Khorana: 1 point each for lung cancer, BMI ≥ 35 kg/m^2^, haemoglobin < 100 g/L, white cell count >11 × 10^9^/L and platelet count >350 × 109/L (baseline biomarkers); (m) PROTECHT: as for Khorana plus 1 point each for gemcitabine chemotherapy and platinum-based chemotherapy; (n) CONKO: as for Khorana excluding BMI and adding 1 point for ECOG PS ≥ 2; (o) CATS: as for Khorana plus 1 point each for sP-selectin ≥ 53.1 ng/mL, D-dimer ≥ 1.44 µg/mL (baseline). BIC, Bayesian Information Criterion; NPV, negative predictive value; NR, not reported; PPV, positive predictive value; ROC, receiver operating characteristic; sHR, sub-distribution hazard ratio.

## Supplementary Materials

The following are available online at http://www.mdpi.com/2072-6694/11/1/50/s1, Figure S1: Cumulative incidence function for thromboembolism and Kaplan Meier estimates for mortality and progression among NSCLC patients receiving ambulatory chemotherapy, according to TE risk classification, Table S1: Biomarker level according to incidence of early clinical events, and by stage and treatment type and intention, for NSCLC patients receiving chemotherapy and/or radiotherapy, Table S2: Expanded biomarker panel among subset of patients treated with chemotherapy with or without radiotherapy.
