# Supplementary material for: Dynamic Thromboembolic Risk Modelling to Target Appropriate Preventative Strategies for Patients with Non-Small Cell Lung Cancer
Source: Cancers (Basel). 2019 Jan 8;11(1):50. doi: 10.3390/cancers11010050 (PMC6356389; doi:10.3390/cancers11010050)
Supplement: Supplementary file 1 [file cancers-11-00050-s001.pdf]

Supplementary Materials: Dynamic Thromboembolic Risk Modelling to Target Appropriate Preventative Strategies for Patients with Non-Small Cell Lung Cancer

Marliese Alexander, David Ball, Benjamin Solomon, Michael MacManus, Renee Manser, Bernhard Riedel, David Westerman, Sue M. Evans, Rory Wolfe and Kate Burbury

A) Study derived TE risk model

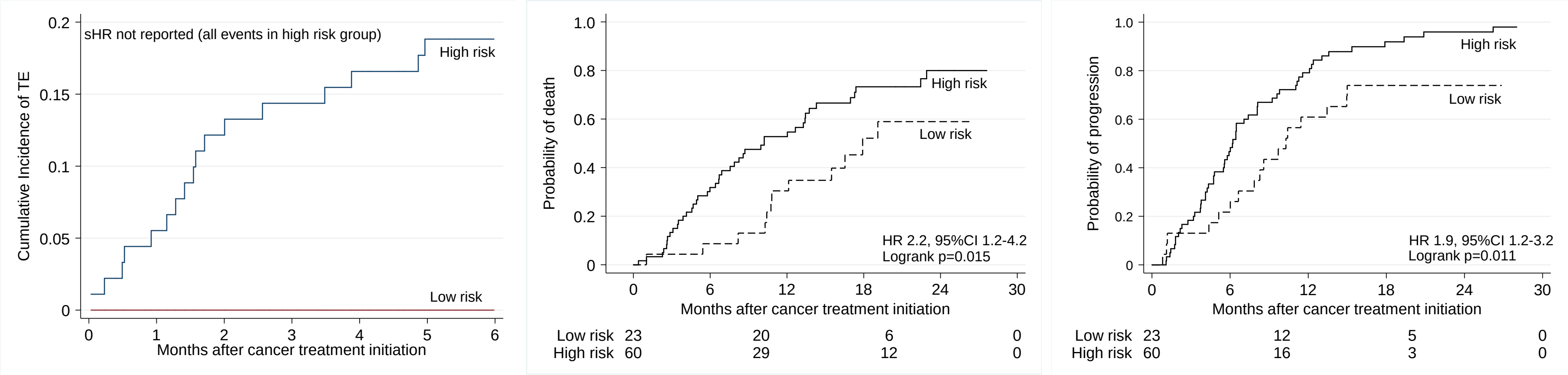

B) Khorana TE risk model

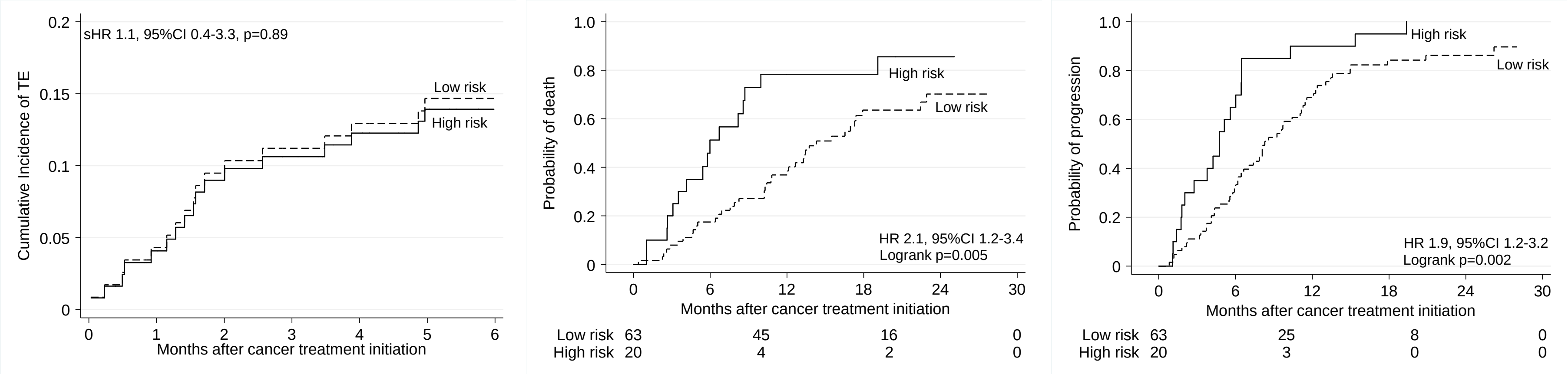

C) PROTECHT TE risk model

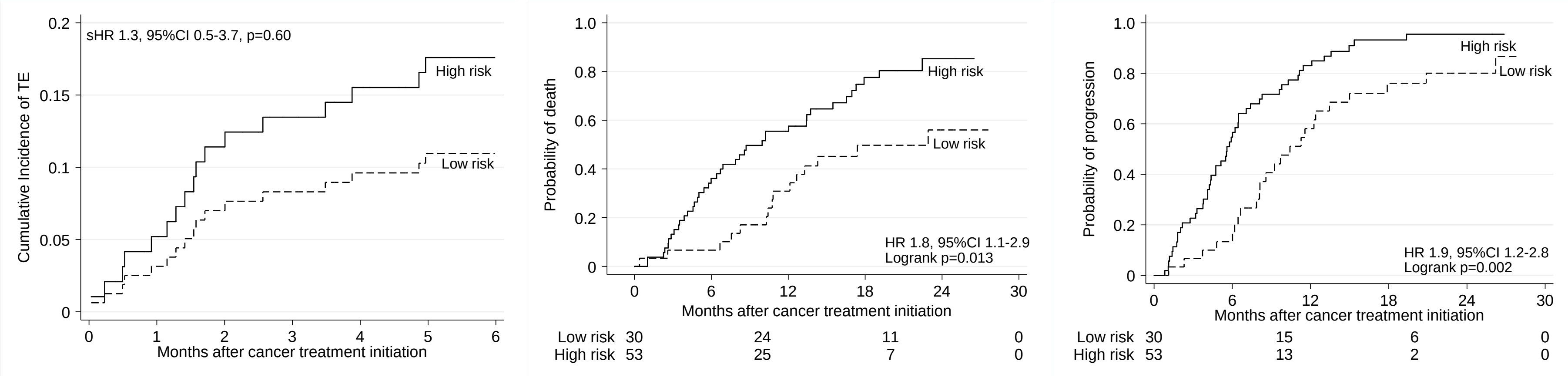

D) CONKO TE risk model

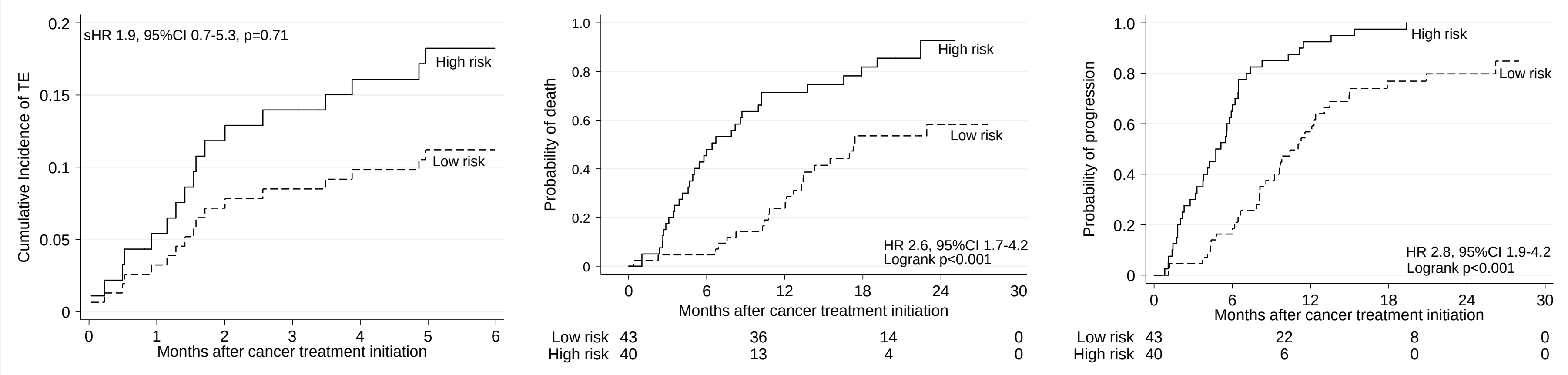

Figure S1. Cumulative incidence function for thromboembolism and Kaplan Meier estimates for mortality and progression among NSCLC patients receiving ambulatory chemotherapy, according to TE risk classification.

Table S1. Median biomarker level according to incidence of early clinical events, and by stage and treatment type and intention, for NSCLC patients receiving chemotherapy and/or radiotherapy.

Timepoint	All Patients Median (IQR), Range [1] (117)	TE ≤ 6 Months			PD ≤ 6 Months [2]			OS < 6 Months [2]			Stage IIIB–IV			Chemotherapy			Curative Therapy			CRT vs. cRT			CHT vs. pRT		
		Yes (16)	No (101)	p [3]	Yes (39)	No (78)	p [3]	Yes (30)	No (87)	p [3]	Yes (68)	No (49)	p [3]	Yes (83)	No (34)	p [3]	Yes (62)	No (55)	p [3]	CRT (47)	cRT (15)	p [3]	CHT (36)	pRT (19)	p [3]
**Fibrinogen—NR 2–4g/L**																									
Baseline	5.3 (4.2–6.2), 2.3–10.7	5.7	5.1	0.56	5.9	5.0	0.01	5.9	5.0	0.02	5.4	4.9	0.17	5.1	5.5	0.04	4.7	5.6	<0.01	4.7	4.9	1.00	5.4	6.2	0.01
Week 1	5.0 (3.8–6.0), 2.2–9.4	4.7	5.0	0.37	5.4	4.7	0.09	5.3	4.9	0.28	5.1	4.8	0.26	4.7	5.7	<0.01	4.4	5.6	<0.01	4.1	4.9	0.20	5.0	5.4	0.01
Month 1	4.9 (4.1–5.7), 2.9–8.0	4.4	4.9	0.46	5.4	4.7	0.01	5.4	4.7	0.03	4.9	4.7	0.35	4.9	4.9	0.61	4.7	5.1	0.09	4.7	4.7	0.81	5.0	5.6	0.41
Month 3	4.9 (4.1–5.9), 2.7–9.4	5.1	4.9	0.96	5.6	4.6	0.10	6.3	4.8	0.07	4.9	4.8	0.64	4.9	4.8	0.89	4.6	5.4	0.22	4.8	4.3	0.18	4.4	5.3	0.28
**D–Dimer—NR<0.5 mg/L[4]**																									
Baseline	0.9 (0.5–1.7), 0.3–8.0	1.1	0.9	0.27	1.1	0.9	0.13	1.3	0.9	0.09	1.2	0.8	<0.01	0.9	1.0	0.35	0.7	1.3	<0.01	0.7	0.7	0.82	1.3	1.4	0.96
Week 1	0.9 (0.6–1.7), 0.3–8.0	1.3	0.9	0.21	1.1	0.9	0.14	1.1	0.9	0.14	1.3	0.7	<0.01	0.8	1.3	0.21	0.7	1.6	<0.01	0.6	0.8	0.18	1.7	1.5	0.42
Month 1	1.1 (0.6–2.1), 0.3–8.0	2.3	1.0	0.01	2.0	0.9	<0.01	2.0	0.9	0.01	1.5	0.8	<0.01	1.1	1.0	0.70	0.8	1.8	<0.01	0.8	0.8	1.00	1.9	1.4	0.35
Month 3	0.9 (0.6–1.7), 0.3–8.0	4.8	0.9	0.08	1.5	0.9	0.13	2.1	0.9	0.08	1.3	0.8	0.02	1.0	0.9	0.88	0.8	1.3	<0.01	0.8	0.9	0.61	1.6	1.1	0.40
**Platelet Count—NR 150–400 × 10^9/L**																									
Baseline	308 (248–414), 88–709	339	306	0.49	340	292	0.03	372	296	0.02	332	282	0.02	308	288	0.87	285	329	0.02	299	270	0.31	317	354	0.28
Week 1	268 (195–346), 82–709	272	268	0.94	279	264	0.85	305	260	0.21	269	264	0.55	237	296	<0.01	268	271	0.82	259	276	0.51	215	356	<0.01
Month 1	246 (183–337), 70–679	252	245	0.91	306	238	0.05	329	240	0.01	279	215	<0.01	245	259	0.49	212	303	<0.01	209	233	0.31	301	303	1.00
Month 3	240 (187–295), 22–673	161	242	0.16	227	241	0.80	226	242	0.80	242	227	0.59	237	251	0.67	237	244	1.00	240	227	0.69	233	255	0.24
**TEG R—NR 2–8 Minutes**																									
Baseline	5.1 (4.5–5.6), 2.8–30.9	4.9	5.1	0.21	5.1	5.1	0.62	5.1	5.1	0.55	5.1	5.1	0.56	5.0	5.2	0.38	5.1	5.0	0.46	5.1	5.2	0.2	5.0	5.0	0.97
Week 1	4.8 (4.2–5.4), 2.8–13.6	4.7	4.8	0.53	4.8	4.8	0.73	4.6	4.8	0.20	4.8	4.8	0.95	4.7	4.9	0.08	4.6	4.8	0.20	4.4	5.3	0.01	4.9	4.8	0.73
Month 1	5.0 (4.7–5.7), 2.3–8.6	5.1	5.0	0.61	5.0	5.1	0.44	5.0	5.1	0.60	5.0	5.3	0.22	5.0	5.3	0.62	5.2	4.8	0.26	5.1	5.4	0.27	4.8	4.8	0.77
Month 3	5.5 (4.8–6.0), 2.5–9.0	5.7	5.5	0.63	5.2	5.5	0.68	5.0	5.5	0.37	5.2	5.7	0.05	5.5	5.4	0.60	5.7	5.3	0.28	5.6	5.7	1.00	5.5	5.2	0.42
**TEG K—NR 1–3 Minutes**																									
Baseline	1.1 (0.9–1.2), 0.8–8.5	1.0	1.1	0.17	1.0	1.1	0.12	1.0	1.1	0.01	1.0	1.1	0.01	1.1	1.0	0.52	1.1	1.0	0.07	1.1	1.1	0.39	1.1	1.0	0.10
Week 1	1.1 (0.9–1.3),	1.0	1.1	0.71	1.1	1.1	0.66	1.0	1.2	0.11	1.1	1.2	0.33	1.2	1.2	0.19	1.2	1.0	0.10	1.1	1.2	0.64	1.2	0.9	0.07

	0.8–8.5																								
Month 1	1.1 (0.9–1.3), 0.8–2.7	1.1	1.1	0.74	1.0	1.2	<0.01	0.9	1.1	<0.01	1.0	1.2	<0.01	1.1	1.1	0.69	1.2	1.0	<0.01	1.2	1.2	0.58	1.0	1.0	0.75
Month 3	1.1 (0.9–1.4), 0.8–2.8	1.3	1.1	0.87	1.2	1.1	0.74	1.1	1.2	0.48	1.1	1.2	0.12	1.2	1.1	0.70	1.2	1.1	0.19	1.1	1.2	0.21	1.2	0.9	0.16
**TEG MA—NR 51–69 mm**																									
Baseline	72 (69–76), 57–85	74	71	0.77	74	72	0.01	75	72	<0.01	74	72	0.01	72	74	0.16	72	74	0.03	72	72	0.44	72	76	0.01
Week 1	70 (67–74), 53–86	73	70	0.76	71	70	0.32	72	70	0.05	71	70	0.22	73	69	<0.01	69	71	0.15	69	71	0.43	69	75	<0.01
Month 1	71 (66–74), 47–82	72	71	0.46	73	69	<0.01	75	69	<0.01	72	69	0.01	71	71	0.95	68	73	<0.01	68	69	0.86	73	72	0.59
Month 3	70 (67–73), 47–82	70	70	0.90	71	67	0.74	72	70	0.36	70	71	0.61	70	71	0.29	69	71	0.53	69	69	0.52	70	73	0.04
**TEG Angle—NR 55–78 Degrees**																									
Baseline	75 (73–77), 31–83	76	75	0.16	75	74	0.07	76	74	0.01	75	74	0.09	74	75	0.61	74	75	0.08	74	74	0.42	75	76	0.12
Week 1	74 (71–77), 46–81	73	74	0.94	74	74	0.75	77	74	0.13	74	73	0.19	73	76	0.06	73	76	0.02	73	73	0.86	74	77	0.06
Month 1	74 (71–77), 54–82	74	74	0.75	76	74	<0.01	77	74	<0.01	75	71	<0.01	74	74	0.90	72	75	<0.01	72	71	0.96	75	76	1.00
Month 3	74 (70–76), 62–79	71	74	0.54	74	74	0.86	75	74	0.37	75	74	0.15	74	74	0.99	73	74	0.34	75	71	0.23	73	75	0.29

[1] Median, interquartile range (IQR) and range of biomarker levels reported for all patients, median biomarker level reported for subgroups; [2] Within six months of anticancer treatment initiation; [3] P-value for the Mann-Whitney test; [4] Laboratory maximum reported d-dimer value ≥8.0. CHT: chemotherapy; CRT: chemoradiotherapy; cRT: curative radiotherapy; IQR: interquartile range; NR: normal range; OS: overall survival; pRT: palliative radiotherapy; PD: progressive disease; TE: thromboembolism.

Table S2. Expanded biomarker panel among subset of patients treated with chemotherapy with or without radiotherapy.

Biomarker [1]	sHR	95% CI	*p*
Thrombin-antithrombin ≥ 4.2mcg/L [2]	1.8	0.7–5.1	0.23
Thrombin-antithrombin ≥ 4.2mcg/L [2]–month 1	7.2	1.6–31.8	0.01
Prothrombin fragments 1+2 ≥ 229 pmol/L [2]	0.9	0.3–2.9	0.93
Factor VIII ≥ 150% [2]	1.6	0.4–7.0	0.56
Fibrin Monomers ≥25 ug/mL [2]	1.2	0.3–5.3	0.84
Phospholipids ≤61 seconds [2]	2.2	0.3–18.5	0.46
Von Willebrand Factor Antigen ≥160% [2]	0.6	0.2–1.7	0.32
ETP ≥ 1930 [3]	9.4	1.2–76.2	0.04
ETP Peak ≥ 260 [3]	9.4	1.2–76.2	0.04
Velocity index ≥ 69 [3]	9.4	1.2–76.2	0.04
Alpha-2-macroglobulin ≥15 [3]	9.5	2.1–43.8	<0.01
ETP ≥ 1930 and ETP Peak ≥ 260 [3]	12.9	1.6–104.6	0.02
ETP Peak ≥ 260 and BIOTEL high risk [3]	11.0	1.4–89.0	0.03
ETP ≥ 1930 or ETP Peak ≥ 260 or Velocity index ≥ 69 [3]	6.9	0.9–56.5	0.07
ETP ≥ 1930 and ETP Peak ≥ 260 and Velocity index ≥ 69 [3]	15.3	1.9–124.1	0.01

[1] All measures assessed at baseline unless otherwise specified; [2] Results available for subset of 74 patients including 14 patients who developed thromboemoblism; [3] Results available for subset of 31 patients including 8 patients who developed thromboemoblism. ETP: endogenous thrombin potential; sHR: subdistribution hazard ratio; 95% CI: 95 percent confidence interval.

© 2019 by the authors. Licensee MDPI, Basel, Switzerland. This article is an open access article distributed under the terms and conditions of the Creative Commons Attribution (CC BY) license (http://creativecommons.org/licenses/by/4.0/).
